# On the Medicinal Effects of the Galvanic Influence, When Employed on Different Parts of the Human Body, or upon Animals during Life

**Published:** 1809-10

**Authors:** 


### *On the Medicinal Effects of the Galvanic Influence, when employed on different Parts of the Human Body, or upon Animals during Life.*

IN that valuable Journal, the *Monthly Magazine,* so respectably known as the vehicle of much *erudition, critical acumen, natural knowledge, and of general science,* a paper was lately inserted, proposing to the humane, a considerably novel means of applying the very interesting, new, and energetic agent, galvanism, from a moderately sized battery, in the resuscitation or restoration to life, of persons apparently drowned.

That this influence is in many respects, and when better understood, perhaps in all, not only analogous to, but actually synonimous with, ordinary electricity, seems to be the general opinion, at the present day, entertained by philosophers and men of cultivated minds, well acquainted with the doctrines of the sciences, and especially attentive to the phænomena of this, which every one apprehends will at no distant day constitute an important pillar both of chemistry and physics, or at least of a very numerous class of the phænomena of nature; and very likely of those arts which having been improved gradually by man, in a favourable condition of society, have so much conduced to its comfort and amelioration, and to that dignified rank which he holds in "*Beings endless chain;* of which he is such a "*distinguished link.*"

We may with justice congratulate the sons of science at the present luminous period, upon the happy results which have attended investigations in this new tract. Not only has electricity and its agencies been ably illustrated by galvanism, but it promises fairly to lay open the sources and origin or foundation of chemical affinities, or the *doctrina corporum individuorum,* and certainly to illustrate in some degree the phænomena of *pathology,* and vitality, in which point of view, what might not be the advantages it will render to the physiologist, and in consequence to mankind.

The elucidation of the subtle principles of latent heat, or the *caloric* of modern chemists, has been admitted to have occurred to at least some extent, and possibly hereafter, and at no very distant period, this hitherto mysterious and *insulated* branch of knowledge, *magnetism*, may not be without analogous illustrations, dependent on a reference to similar means.

The identity of light, caloric, and electricity, have been shrewdly conjectured, by some men whose opinions are held in esteem and viewed with reverence, because they are connected with the well earned fame such characters have acquired by successful labours in this field, in which still so much remains to be done.

That GALVANISM and ELECTRICITY are considerably analogous, has been apprehended, not merely from the correspondence of the effects produced upon the recent limbs of animals, after amputation, and especially in such as are termed cold blooded, when in contact with, or in the near neighbourhood of the *torpedo, gymnotus electricus*, or *eel* of *Surinam*, together with many others of the same tribe, and which announces such a correspondence between *animal* and *ordinary electricity* and *galvanism*. Tho' the effect of the *shock*, as it is generally termed, be perhaps merely in each instance, a modification of the same primitive great principle; yet the effects of common electricity and the galvanic energy are conspicuous in their energies upon inorganic matter.

This is proved by the usual and well known attendant sparks, corruscations, and phosphoric illuminations, the kindling of most combustible bodies, as carbon, phosphorus, sulphur, the metals, and not merely such as have been heretofore, by the elder chemists, termed *imperfect, ignoble*, or *semi*, (because alterable by exposure to fire, air, moisture, or acids) but also from the effects of these most powerful agencies upon the *noble* metals, such as gold, platina, &c. which were not acted upon by the above means under ordinary circumstances. Not to mention the deflagration which in both cases occur, when these influences are made to pass, by suitable conductors, through mixtures of the highly oxygenated and readily decomposable salts, as the hyper-oxymuriate of potash, the oxymuriate and nitrate of the same alkali, or of soda, or of the earths or metals. In these last instances, effects are produced *in vacuo*, similar to those presented in the open air, and especially in pure pure oxygen gas or vital air, for which purposes, gold or platina wires are preferably employed.

Hence imitations, much admired and very instructive, of *volcanoes, thunder, lightning*, the *aurora borealis*, and of other natural and astonishing phænomena, are exhibited and illustrated in the schools of chemistry.

And it were very much to be desired, that more attention had been paid, and more experiments made, upon animal electricity, for by analogy and comparison much is learned in the inductive sciences, and certainly such as rest upon experiments are so. The torpedo ought to be the subject upon which the train of inquiry I allude to should be founded. I will not here deny that valuable results have attended the curious anatomical labours of a Hunter, or the ingenious, if not correct, reasonings or theories of Volta or of Galvani. But it may be permitted to me to hope, that pursuits, in which the very obvious good of the human race is much concerned, will not cease to be inviting. The endeavours to gratify an honourable and praiseworthy curiosity, have their use—still, *experimenta desiderantur*. And whoever shall seriously set about cultivating this promisingly prolific field, will be gratified by an abundant harvest. It is matter of much gratification to us to know, that the needful apparatus are far from costly, and that with the labours of the learned, as far as they have yet proceeded, every publication teems, from the thin folio of a common ephemeral Journal, to the more voluminous and erudite transactions of philosophers.

I know not, Mr. Editor, when treating on a subject of such magnitude, almost where to pause, nor indeed precisely, when or where to begin.

Nothing can be more obvious than that the galvanic influence, passed through parts chronically affected by disease, must induce some considerable change in them, when we know from the instructions of Aldini, and our own experience, that the most horrible contortion and grimaces are excited in the subject recently deceased, and especially since we have been, by fatal occurrences, made evidences of the consequences which the electrical fluid produces when passed through the more sentient parts of the system.

Is not this capable of happy illustration by the following simple, yet well known and satisfactory experiment.

Exp.—Pure distilled water, at such temperature as excludes all possible presence of azot or nitrogen in solution, carefully introduced into a tube of inconsiderable diameter, and and moderately strong, and to which two small wires of pure gold, approaching each other within half an inch, and which are previously passed through perforated corks or rather wax, will be decomposed when their other terminations touch the two poles of a galvanic battery of moderate size, say of fifty successive series or pairs of plates, (of course the larger the more speedily is the effect accomplished.)

As soon as the contact is made, a rapid disengagement of hydrogen and oxygen gases respectively, and at the different terminations of the wires within the water, is abundantly perceptible. A quantity of water is decomposed, and disappears equal in weight to the weight of the gases disengaged. It is true that these gases will be held in solution in part, and in a very strong vessel might possibly in their nascent state again combine; but the sides of the containing vessel will sooner give way by the elastic force of the air, *ex interno,* where thin glass is employed.

*Note.*—The writer is not unaware of the production of water by the mechanical compression of its constituent gases ; but if so then an inordinate force is requisite for this purpose, and a much more powerfully resisting apparatus than that which is here alluded to.

The above experiment may be performed by an electrical machine. Indeed, the effect was first accomplished of decompounding water by electricity, with the powerful apparatus of Dr. *Van Marum,* at *Haarlem,* in the presence of several Dutch chemists of high celebrity, which we believe is well known to every chemical philosopher in this country.

But it is the application of this property of the galvanic fluid or electric energy to which we would call the attention of your numerous readers; and that the conclusion is just which we do draw, anatomical dissections after death will prove.

For the production of gases from any, or all the animal fluids, when inclosed hermetically, and operated upon in the same manner as the water in these glasses, is still more obvious ; and there can be no doubt but that when death is induced by passing strong shocks through the brain, by attaching galvanic conductors, and using powerful machines, or a considerable number of discs of copper and zinc in the batteries ; that if death be the consequence, it will be from oppression, or compression of the brain by the extravasation and consequent affusion of fluids from their proper vessels upon the brain itself; these vessels being being ruptured, as glass is fractured by the disengagement of air on the decomposition of the contained fluids. It might be urged that the elasticity of the sides of the vessels of the human frame might hinder this effect, but their resilient faculty may not (under the circumstances of very powerful shocks) be equal to the necessary resistance; and upon these grounds, as well as because all experience teaches the consequence of too powerful shocks, we would recommend a practice different from that employed in cases when electricity has been indicated; and yet probably more efficacious in the end, certainly more grateful to the patient, and beyond all comparison less troublesome to the medical superintendant, whose time and engagements preclude him from directing the most obvious and most powerful means, the means of electricity.

"When men are united in a state of improved society, and have been in habits of enterprize of common concern, the necessity of a rapid and distinct communication of ideas will make a forcible impression on them." Why the bee and the beaver so much cultivate the cares of a common-wealth, is as obvious as the adage itself is trite. In this duty we are unwilling to be last.

In what follows, as well as what has been said, some may find redundancies and omissions, and other deficiencies and imperfections; but there is one point on which there will be no diversity of sentiment, the importance of the subject, especially to that class of society whose labours, toils, and study, devote them professionally to succour the sick, and attempt relief when hope has almost deserted the deplorable objects of their good intention to relieve.

The galvanic current admits of easy application to almost any parts of the body, and will enable us doubtless, hereafter, to understand well the doctrine of consent between such as are most distant and apparently unconnected, or which the anatomist can find no particular reasons for considering as more especially connected by nerves or otherwise, than with other distant parts generally. Thus, in *indolent* tumours, a gentle current of electric matter has been often recorded as attended with effects most beneficial. The wires need only be prescribed at a moderate distance from the tumour, or diametrically opposite, and so as that the tumour should be strictly interjacent. Or even the conductors connected with the opposite poles of a galvanic pile or battery, may be presented to the base of the tumour on each side, or as the circumstances and conditions of the case naturally enough suggest, or as a medical attendant, acquainted with all concurrent circumstances, would advise. All that is farther requisite should seem to be, that no higher or more intense a stream of electric fluid is to pass the tumour than can be well and without particular pain borne. Indeed, in many instances, as in cases of *hydrocephalus internus*, in infants or of those of more tender age, and who are too well known to be the frequent victims of this perverse malady, the influence might be applied between the mouth and *occiput*, by closely attached and connecting wires with a small battery at the foot of the bed while they are still asleep. It must be obvious that a very small power need only be here applied. The object is not to prevent extravasation or organic lesion of any containing vessel, but to create activity in the more torpid absorbents, by means whereof, accumulated and offending matter may be removed. For what are medical artists more than the accoucheurs of nature? It would be needless to advise early application, the importance of all remedial powers must be recommended as soon as these powers are indicated by the proper indices which characterize respective diseases.

*Principiis obsta, sero medicina paratur,*

*Cum mala perlongas in valuere moras,*

*Sed propera nec te venturas differ in horas,*

*Qui non est hodie, cras minus aptus erit.* OVID.

And our English couplet to the same purpose:

The young disease which must subdue at length,
Grows with our growth, and strengthens with our strength.

Independent of the agency of external powers, we know that the mind itself has no inconsiderable influence, under particular circumstances, in determining the flux of blood to different parts, and produces thus in the pervasion of vessels, usually empty or not inordinately filled, a considerable and distinct effect. The lively red of the cheeks, suffused by the bloom of health, or induced by delicacy, connected with energy of thought or great sensibility, shews that this part of the skin varies in its properties from others, which, under the same impressions, do not undergo similar changes. The same immediate causes do not alike produce it in all persons, though the remote or ultimate means must be the same in all, as certain affections of the mind; such as joy, shame, &c. The arterial vessels which assist in composing the internal fabric of the *cheek*, are larger and much more numerously dispersed than in other parts. The cheek has been compared to a beautiful piece of fillagree, wherein the minute capillary arteries, fine hair-like filaments ments branch out, and are variously interwoven in numberless ramifications, which imperceptibly diminish until they terminate in colour, tint, and healthy bloom upon the surface. This curious mechanism is invariably the same in all; and if it does not produce the same lively hue in each, we must attribute the effect to obstruction, construction, or diminution of size of the vessels from some other cause.

But to return.—From what has been said, the superior simplicity in the application of the galvanic energy, capable of being made imperceptibly efficacious, or of being, as to its intensity, incalculably extended, (should occurrences demand it) no comparison exists between its power thus applied and that of common electricity, excited by ordinary machines; for the Leyden phial once discharged, exhibits no more power until renewed by continued excitation and recharge, whereas the galvanic electricity, as we may venture to term it, seems to accumulate a strength so gradually diminishing, as to be almost imperceptible after the conclusion of the most needfully protracted operation. In addition to which, the patient himself can in most cases be put into possession of the means of continuing in many chronic affections, the operation, and of lessening or increasing the intensity of the agent, according as he may be inclined; and in these cases his feelings will be his best, most faithful, and most unerring guides.

Moreover, it has been asserted, that when the vigilant powers of the mind are suspended, as during sleep, all external agents, and especially those which are connected with muscular energies, excite an increased action; admitting this to be true, and that while the body is obedient to the will, as when we are awake, a sort of offset to the energies of extraneous and especially unusual stimulants is made; yet it should not be forgotten, that as gradually will the powers of the battery diminish. We are, however, under the necessity of supposing, that the imagination has indulged itself too far when these conclusions have been deduced from the effect of the Leyden jar, when discharged through a paralytic limb, which of the same intensity, is said to be greater as to muscular energy during sleep.

Pictet, who has recorded some very valuable and successful applications of electricity at *Geneva*, upon patients (when all other means had failed) expressly declares it to be his decided opinion, that it had in none in which

 he he had superintended its energies, known it to succeed, except when small shocks were continued, and that for a considerable length of time.

"*Gutta cavat lapidem non vi sed sæpe cadendo.*" But an immense number of small shocks, or a continued but moderate and *tolerable* stream of galvanism being employed, produces a much greater effect, and in some instances speedily, as the following fact evinces.

The writer witnessed, in the presence of ——— Becher, D. D. of *Southwell,* near Mansfield, the sudden effects of the galvanic influence when passed through the eye, by bringing the conductors of a moderate battery into contact with the *recti muscles,* and, indeed, alternately passing the *aura,* which was as often as the patient, a girl, could bear it interrupted so as to give shocks. She could not, when the operation commenced, distinguish a radish which was in a sallad dish on the table; but in less than three-quarters of an hour, occasionally resting, she actually threaded a small needle, presented to her for that purpose by the Doctor's lady, who with several clergymen of that place were present. She had been taken as a poor girl to work at a neighbouring cotton mill, with a view to take up such ends and piece them, as from time to time broke in her part of the spinning machinery. She was turned away for doing more harm than good at the mill, in this calling, to which from the material deficiency above alluded to, she had been for some time exposed. It is now some time ago, as much as three years; she may have since had a relapse, but this is not known to the writer.—Her age was about 14.

The cases, in which this well-known stimulus may be expected to succeed, will be obvious from a knowledge of many cases of electric agencies, to the expert physican. For it will be too much to expect that it should be employed with safety or success in all; nor must it be apprehended, that the tractors, (which have been upheld, not merely by maniacal impostors, but the most artful, expert, audacious and interested quacks) have any such powers; indeed, they can have none, unless through the medium of imagination upon the minds of the debilitated and the feeble, in whose nervous *fancies* constantly hovers the image of agony—of agony unsuspected—and which has consigned them as a too easy prey to an artifice of which they might possibly become the unhappy victim. But it is to be hoped delusion has vanished here, as it has from the wiser children of the New World long ago, and that Perkinism and and Mesmerism are no longer protected by the laws of any enlightened land.

From defect of energy of the brain, or from loss of tone of any of the organs of sense, as the olfactory, aural, palatal, or optic nerves, the galvanic influence, applied agreeably to our instructions, may be of obvious utility; but it must always be presumed, that no organic læsion has occurred in any instance, at least, where this power is expected to be efficacious. In paralytic cases indeed, it promises much success. From what has been already said, it will be held as doubtful in apoplexy. Indeed, it is not easy to see of what use it could be here. Some think, where there is not any præternatural determination to the head, or accumulation of blood in the vessels of the brain it may be attempted with safety. To decide in these instances, is, perhaps, very considerably difficult; and to advise, when we do not see the ground clear, is to inculcate a conduct which might lead to personal disrepute in practice; and the prevention of valuable applications of an interesting and powerful remedy, where such remedy is in reality indicated.

The cuticle, when slightly removed by blister or otherwise, gives a much more perceptible effect to a moderate pile or small number of series of the metals respectively employed for exciting the effect; but this mode is little used from the unpleasant sensations excited on the parts when the conductors are presented.

It is very likely, that in phonology, when the organs of speech are without deficiency, except of nervous energy, as in cases of stammering, galvanism might be applied with advantage, by passing a current through them as already directed in other cases. At least, we think this worthy the attention of those who profess to cure the deficiences of articulation, which often proceed from ill contracted habits, or from general or local debility. But this is merely a hint, which will be, perhaps, advantageously taken by those who engage themselves on this occasion with industry and penetration. There may be other causes of the "*Vox faucibus hæsit.*"

A Case is recorded, in which it is stated, that by passing repeated shocks between the nape of the neck and the tongue, the party succeeded in restoring the organs of speech to a person who could not express a word so distinctly as to be understood; the operation was accomplished in a few minutes, and it is not stated that any pain was attendant on the process. We understand, that at Edinburgh,

Edinburgh, there are actually stammering schools—Query, Has this principle been yet employed, either there or in London, with the above view?

In paralytic limbs, when the wasting has not been considerable, and especially in the more recent cases, the pallid appearance is succeeded by a considerable rubefacience, becomes often intumescent, accompanied by a tingling sensation, with gentle, and in some instances, very considerable perspiration, all of which are to be accounted favourable omens; clinched fingers, and other contractions, have in like manner given way. Œdema is often seen to disappear, by the adhibition of moderate shocks, and thus occasionally a favourable termination, with the aid of few auxiliary internal means, except indeed such as are naturally indicated by temporary circumstances, are required.

It has been thought to be of probably much value, as an application in cases of tetanus or lock jaw; and for dispersing gouty rigidities, or such as are consequent to gout in joints. It is very desirable that the cases of lock jaw should be attended to in this way.

Whether any, and what, might be its effects in particular cases, such as cancer and white-swellings, as they are termed, we yet remain to learn.

Whether a nerve be alive to the stimulus, especially of galvanism, may be ascertained by trying whether the muscle can contract or not. Thus, in amaurosis, if the nervous flash is not perceived when the conductors pass from the apparatus to the recti muscles of the eye, it is obvious that no human art can re-produce the blessing of sight.

In the common electrical experiments, if a paralytic patient make part of a ring or circle, through which the Leyden phial is attempted to be discharged, it will be found impossible to do so, when the limb is completely deprived of its nervous energy, so sensible is the fibre to the stimulus so calling forth its energy.

In chronic cases we have said that its uses are great; and even in chronic rheumatic affections it has its value; but in acute cases, what can be expected from it? One would *a priori* imagine, that it would not at any rate diminish the malady.

Galvanism is thought to afford a valuable diagnostic, in cases where we cannot by the pulse or other means, distinguish whether rheumatism be acute or chronic. In the former case, it never fails to add to the irritation and painful painful symptoms, which are deeply diminished by it in the latter.

In cases of amenorrhœa, it is well known that electricity has been employed with the most favourable results; and if so, galvanism must be especially recommended.

For the same reason, it is worthy of trial in obstructions of the liver, and complaints arising in that region. We hope to be enabled speedily, to furnish farther details on this subject.

In diseases of the mind, galvanism has been said to have been, when judiciously employed, particularly in *melancholia*, attended with success. Very many opportunities unfortunately present themselves to ascertain these facts. Why have we not any accounts of the experiments which ought to have been made, especially after the successful report of *Aldini?* These diseases are deeply connected with such as are corporeal; it is to be lamented that they have not been more studiously attended to.

That physiologist, who undertakes to continue and extend the principles of modern electro-chemical science, as galvanism has been very properly termed by the ingenious Professor Davy, will deserve well of the laudable cause of humanity. The chemical physiologist need not now be informed of the importance it would be to medical science, to bring every hypothesis to the test of severe experiment; for chemistry is at the present day, different indeed from what it was at that unfortunate period, "when the extravagance it had introduced into medicine made the alliance dangerous." The method of the immortal Bacon should be employed in all cases; "where we would endeavour to rectify the mind, guard it against error and illusion, and conduct it to the fountains of Nature and of use." It is thought, that if this practice were more generally pursued, the business of invention would not long continue that casual thing it is; but be reduced to an art, which of all others is perhaps most wanted."

I shall abruptly conclude this Essay, in the language of *Solon,*

Ἔργμασιν ἐν μεγάλοις πᾶσιν ἁδεῖν χαλεπόν.

———*Omnibus in magnis difficile ut placeas.*

LOCK

